# STN Versus GPi Deep Brain Stimulation for Action and Rest Tremor in Parkinson’s Disease

**DOI:** 10.3389/fnhum.2020.578615

**Published:** 2020-10-23

**Authors:** Joshua K. Wong, Vyas T. Viswanathan, Kamilia S. Nozile-Firth, Robert S. Eisinger, Emma L. Leone, Anuj M. Desai, Kelly D. Foote, Adolfo Ramirez-Zamora, Michael S. Okun, Aparna Wagle Shukla

**Affiliations:** ^1^Department of Neurology, Normal Fixel Institute for Neurological Diseases, University of Florida, Gainesville, FL, United States; ^2^Department of Neurosurgery, Normal Fixel Institute for Neurological Diseases, University of Florida, Gainesville, FL, United States

**Keywords:** deep brain stimulation, tremor, parkinson disease, STN, GPi

## Abstract

**Objective:**

To investigate the effects of subthalamic nucleus (STN) and globus pallidus internus (GPi), deep brain stimulation (DBS) on individual action tremor/postural tremor (AT) and rest tremor (RT) in Parkinson’s disease (PD). Randomized DBS studies have reported marked benefit in tremor with both GPi and STN and DBS, however, there is a paucity of information available on AT vs RT when separated by the surgical target.

**Methods:**

We retrospectively reviewed the 1-year clinical outcome of PD patients treated with STN and GPi DBS at the University of Florida. We specifically selected patients with moderate to severe AT. Eighty-eight patients (57 STN and 31 GPi) were evaluated at 6 and 12 months for changes in AT and RT in the OFF-medication/ON stimulation state. A comparison of “response” was performed and defined as greater than or equal to a 2-point decrease in tremor score.

**Results:**

STN and GPi DBS both improved AT at 6- and 12-months post-implantation (*p* < 0.001 and *p* < 0.001). The STN DBS group experienced a greater improvement in AT at 6 months compared to the GPi group (*p* = 0.005) but not at the 12 months follow-up (*p* = 0.301). Both STN and GPi DBS also improved RT at 6- and 12-months post-implantation (*p* < 0.001 and *p* < 0.001). There was no difference in RT scores between the two groups at 6 months (*p* = 0.23) or 12 months (*p* = 0.74). The STN group had a larger proportion of patients who achieved a “response” in AT at 6 months (*p* < 0.01), however, this finding was not present at 12 months (*p* = 0.23). A sub-analysis revealed that in RT, the STN group had a larger percentage of “responders” when followed through 12 months (*p* < 0.01).

**Conclusion:**

Both STN and GPi DBS reduced PD associated AT and RT at 12 months follow-up. There was no advantage of either brain target in the management of RT or AT. One nuance of the study was that STN DBS was more effective in suppressing AT in the early postoperative period, however, this effect diminished over time. Clinicians should be aware that it may take longer to achieve a similar tremor outcome when utilizing the GPi target.

## Introduction

The cardinal motor features of Parkinson’s disease (PD) include resting tremor, bradykinesia, rigidity and postural instability. Although resting tremor (RT) is one of the most notable features of PD, action tremor (AT) is commonly encountered. Studies estimate that as many as 46 to 92% of PD patients will develop AT at some time during their disease course (Louis et al., 2001; Kraus et al., 2006; Gigante et al., 2015). While RT has a significant impact on the quality-of-life and can be debilitating especially in social situations, AT can interfere with the ability to execute motor tasks (Zimmermann et al., 1994; Forssberg et al., 2000; Louis and Machado, 2015).

Deep brain stimulation (DBS) is an established therapy for the treatment of motor symptoms in PD and has been shown to be more effective than best medical therapy in improving motor function and quality of life in well-selected PD patients (Weaver, 2009). The subthalamic nucleus (STN) and the globus pallidus internus (GPi) are the two most frequently used FDA approved brain targets for the management of medication refractory PD tremor. Several studies have compared the effects of these targets on the combined control of RT and AT (DBS for PD Study Group, 2001; Anderson et al., 2005; Weaver et al., 2012; Odekerken et al., 2013). Recently, our group published a meta-analysis on this topic and found no significant differences in tremor control between the two targets (Wong et al., 2018). Nonetheless, there is a lack of information in the literature specifically describing the longitudinal effects of DBS on AT or in comparing brain targets. A recent retrospective study found equivalent tremor outcomes, however, DBS targets for comparisons involved STN and the ventral intermedius nucleus (VIM) but not the GPi (Parihar et al., 2015). In the current study, we evaluated the longitudinal tremor outcomes in PD patients with moderate to severe AT managed with either STN or GPi DBS. Additionally, we investigated whether pre-surgical or other factors could affect AT tremor outcome.

## Materials and Methods

### Patient Selection

The study was approved by the institutional review board at the University of Florida. We extracted data from a longitudinal research database for a retrospective analysis of tremor outcomes in PD patients receiving DBS surgery at our center from 2004 to 2016. The inclusion criteria for the study comprised the following (1) Diagnosis of PD was established with United Kingdom PD Society Brain Bank Criteria (Hughes et al., 1992) (2) Patients had moderate to severe AT before surgery corresponding to a score of ≥2 on item 21 the Unified Parkinson’s Disease Rating Scale (UPDRS) part III. (3) Unilateral or bilateral DBS of either STN or GPi nucleus. If the patient received bilateral DBS, we included data only for the most affected tremor side. (4) Patients in the two comparison groups (STN vs GPi) had 12-months follow up data. (5) Patients accomplished optimal programming parameters for the implanted lead within 4–6 months of surgery. The exclusion criteria consisted of (1) Baseline assessments before surgery not documented (2) During the postoperative follow-up, the patient was deemed to have a suboptimal lead placement (3) Patient had prior neurosurgery for PD (4) The patient received a diagnosis of an atypical parkinsonism syndrome any time during follow-up after DBS.

### Standard Perioperative Procedures Followed for All Patients

Parkinson’s disease patients underwent a multidisciplinary team evaluation consisting of neurology, neurosurgery, psychiatry, neuropsychology, and rehabilitation disciplines. Upon completion of the discussion of the benefits and the risks, DBS surgery was scheduled. Based on our experience from the COMPARE trial and available literature, patients at our center are generally recommended GPi DBS when they report prominent levodopa-induced dyskinesia or when they exhibit concerning cognitive or mood difficulties. The STN target is usually recommended for patients with debilitating tremor, prominent akinesia, rigidity or those who are experiencing prominent dopaminergic medications adverse effects without significant cognitive impairment or dyskinesia (Pollak et al., 2002; Okun and Foote, 2005; Okun et al., 2009; Tagliati, 2012; Williams et al., 2014). However, as multiple factors play a role in target selection, we identified multiple patients in our database managed with GPi DBS despite the presence of significant tremors. Patients receiving bilateral DBS at our center are operated in a staged fashion with approximately 6 months in between lead implantations.

On the day of the surgery, an atlas-based anatomical mapping of the target location was performed on a preoperative CT scan fused with a 3T MRI image (Sudhyadhom et al., 2012). Further guidance for lead implantation was obtained from the intraoperative microelectrode recordings and macrostimulation testing performed in the operation room immediately after the lead was implanted. The DBS lead (model 3387; Medtronic, Minneapolis, MN, United States) was implanted under local anesthesia and for confirmation of lead location, a postoperative CT scan was performed and fused with the preoperative MRI image. Pulse generator implantation (Activa, Soletra, or Kinetra; Medtronic, Minneapolis, MN, United States) surgery was scheduled within 2 weeks of lead implantation surgery. Patients were followed at regular time intervals (every month for the first 6 months, and every 3–6 months for the second half of the first year); they underwent standard procedures for optimization of stimulation settings and adjustment of medication doses.

### Outcome Measures and Longitudinal Follow-Up

Patient demographics, perioperative DBS information, and UPDRS scores were collected for patients in each group. Pre-DBS baseline UPDRS scores were collected in the OFF medication state and the post-DBS scores at around 6- and 12-months follow-up were collected in the OFF medication/ON stimulation state. A 2-months margin was applied for follow up at each of the time intervals, e.g., the 6-months post-implantation time point included visits from 4 to 8 months post-surgery. Dopaminergic medications were withdrawn for a minimum of 12 h for the OFF medication state assessment. We had two patient groups for comparisons (STN DBS vs GPi DBS).

We determined the severity and occurrence of AT in the contralateral arm based on item 21 of the UPDRS part III assessment. We further extracted contralateral side RT (item 20), bradykinesia (summation of items 23–25 for the upper extremity), rigidity (item 22 for the upper extremity) and total motor score (summation of items 18–31). Compared to baseline, the UPDRS change in AT, RT, bradykinesia, rigidity, and total motor score at 6 and 12 months after surgery was calculated for each of patient groups. The primary outcome for the study was the change in AT score at 6 and 12 months compared to baseline in the STN group versus the GPi group. Secondary outcomes analysis included the comparison of changes in RT, bradykinesia, rigidity and total motor scores at these follow-ups. The secondary outcomes also included comparisons of RT vs AT outcomes for each of the targets and the baseline factors impacting these outcomes at 6 and 12 months.

### Statistical Analysis

The clinical data was imported into IBM SPSS Statistics 26 software for statistical analysis. We compared the demographics, baseline clinical measures, and UPDRS scores between the two groups using Mann Whitney *U*-tests, Pearson’s Chi-squared test or Fisher’s exact tests, as appropriate. We set the statistical significance to a threshold of *p*-value < 0.05. We utilized non-parametric tests for analysis, considering the small sample size and non-normal distribution. The Wilcoxon signed-rank test was used to compare the DBS effects on AT and RT at 6- and 12-months follow-up compared to baseline. A Bonferroni correction was utilized for multiple comparisons, assuming alpha <0.05 and two statistical analyses, corrected *p* was <0.025. For the between-group (STN vs. GPi) comparison of the change in outcome measures at 6- and 12 months follow-up compared to baseline, we employed a Mann Whitney *U*-test with Bonferroni corrections as previously mentioned.

In the STN and GPi groups, we also identified the “optimal responders” and “suboptimal responders” based on ≥2 or <2-point change in AT or RT score at each of the follow-ups compared to baseline. While all patients in our group at baseline had AT score ≥2, whether there was change in RT ≥ 2 or <2-points was determined in only those patients with a baseline score = 2. We further examined in a binomial logistic regression model whether age at surgery, duration of disease, baseline motor severity, baseline tremor severity, levodopa responsiveness of total motor score, and levodopa responsiveness of AT or RT (depending on AT or RT outcome) influenced the >2 point decrease in the tremor score at 6 and 12 months follow-up. Levodopa responsiveness for RT and AT was ascertained separately and was defined as a >30% decrease in the on-medication score compared to off-medication score. The variance inflation factor (VIF) and tolerance were calculated for all model variables to test for multicollinearity. A VIF threshold of 4 and tolerance threshold of 0.2 was used for the regression model (Hair et al., 2009).

DBS contact locations were measured from a post-operative non-contrast CT head. During pre-operative targeting, modified and digitized Schaltenbrand-Bailey atlases (AC-PC space) were manually fitted to each individual’s pre-operative MRI brain using a linear transformation by the neurosurgeon (Sudhyadhom et al., 2012). The two images were then co-registered and reverse normalized into the AC-PC space for comparison across patients. Calculations were performed using MathWorks MATLAB R2016b. We considered the location of an active contact to be the center position of the contact for monopolar settings or the mean position between multiple contacts for bipolar settings. The locations of the active contacts were then visualized as three-dimensional spheres centered at the mean location of the active contacts and with radii given by the standard error of the active contacts. This was further superimposed upon the digitized Schaltenbrand-Bailey atlas. The size of the sphere does not represent the DBS electric field or volume of tissue activated. In this visualization we separated “optimal responders” versus “suboptimal responders” and we separately analyzed AT versus RT and STN versus GPi groups. The left and right hemispheres were collapsed for statistical comparisons. We compared the *x*, *y*, and *z* coordinates of suboptimal versus optimal responders using a *t*-test or Wilcoxon-signed rank test for normal or non-normal data, respectively, which was assessed using the Shapiro-Wilk test.

## Results

**FIGURE 1:**
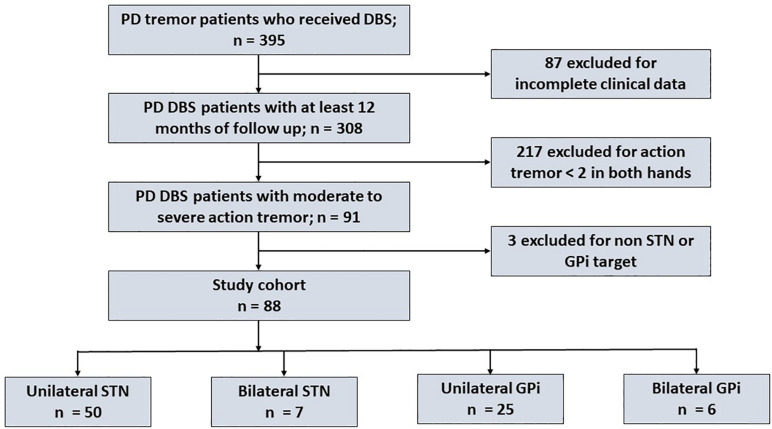
Flow chart illustrating the patient selection process for our study cohort.

We identified 395 PD tremor patients who received DBS at a single center, however, due to various reasons including a baseline AT score that was <2, incomplete data at follow-up assessments and DBS targets other than STN and GPi, we excluded 307 patients. As shown in Figure 1, 88 PD patients (57 STN, 31 GPi; 68 males, 20 females) were included in the final cohort. Most patients had unilateral DBS with *n* = 50 unilateral STN, *n* = 25 unilateral GPi, *n* = 7 bilateral STN and *n* = 6 bilateral GPi. The mean (±SD) age was 61 years (±9.7; range 39 – 81 years), the mean disease duration was 12 years (±5.6; range 2 – 30 years), the mean baseline UPDRS III motor score was 45 (±11.1; range 16 – 77), the mean baseline AT score was 2.4 (±0.4; range 2 – 4) and the mean baseline RT score was 2.5 (±1.1; range 0 – 4). The mean total motor, AT, RT, rigidity and bradykinesia scores were not significantly different between the two targets. As shown in Table 1, except for the disease duration that was significantly longer in the GPi DBS group (*p* = 0.006) compared to the STN group, the baseline variables were not significantly different.

**TABLE 1:** Baseline characteristics of Parkinson’s disease patients receiving STN and GPi DBS.

**Patient demographics (*n* = 88)**	**STN (*n* = 57)**	**GPi (*n* = 31)**	***p*-value**
Sex, *n* (male%)	46 (80.70)	22 (70.97)	0.30
Handed, *n* (right%)	48 (84.21)	27 (87.09)	0.72
Age, years (SD)	61 (10.33)	63 (8.12)	0.41
Disease duration, years (SD)	11 (5.02)	15 (5.85)	0.01
Bilateral DBS leads, *n* (%)	7 (12.28)	6 (19.35)	0.38
Levodopa responsiveness, *n* (%)	41 (71.93)	17 (54.84)	0.28
**Baseline UPDRS scores**			
Total motor score (SD)	44.12 (10.45)	47.32 (11.79)	0.20
Action tremor (SD)	2.42 (0.65)	2.29 (0.45)	0.33
Severe (score 4), *n*	5	0	
Moderately severe (score 3), *n*	14	9	
Moderate (score 2), *n*	38	22	
Rest tremor (SD)	2.68 (1.05)	2.26 (1.14)	0.08
Rigidity (SD)	1.16 (0.81)	1.54 (0.63)	0.14
Bradykinesia (SD)	2.18 (0.76)	2.46 (0.49)	0.23

*STN, subthalamic nucleus; GPi, globus pallidus internus; DBS, deep brain stimulation; SD, standard deviation. Levodopa responsiveness is defined as greater than 30% reduction of UPDRS part III total motor score.*

### DBS Response for Action vs. Resting Tremor and Comparisons Between Targets

**FIGURE 2:**
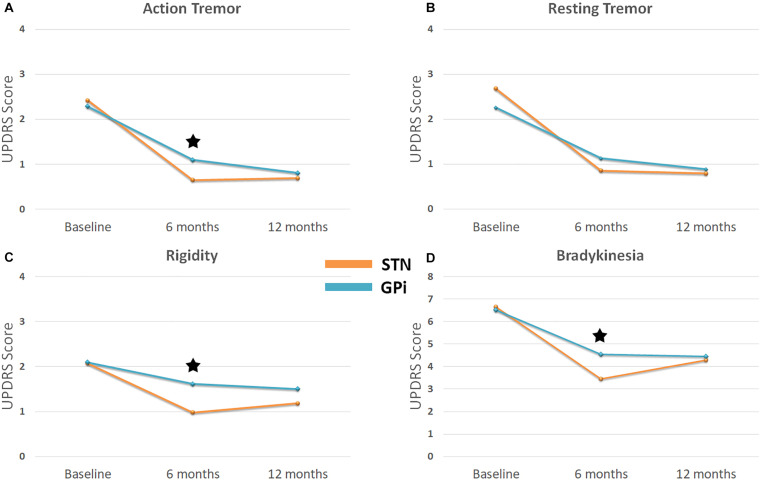
Line graph illustrates outcomes for **(A)** action tremor **(B)** rest tremor **(C)** rigidity and **(D)** bradykinesia for STN DBS versus GPi DBS. Blue line represents STN DBS and orange line represents GPi DBS. Baseline represents UPDRS item scores before surgery. UPDRS item 20 applicable to contralateral arm was used for assessment of rest tremor, item 21 for action tremor, item 23 for rigidity and summation of items 23, 24, and 25 for bradykinesia. Scores at 6 and 12 months after DBS were obtained in during OFF medication – ON stimulation state. *Denotes a statistically significant difference between the two groups.

The mean baseline AT scores for STN and GPi groups were (2.42 ± 0.65) and (2.29 ± 0.46), respectively. Compared to baseline, the mean score and percentage improvement at 6 months follow-up were significant for STN (0.65 ± 0.86; 73%, *p* < 0.001) and GPi (1.09 ± 0.83; 52%, *p* < 0.001) groups. Furthermore, compared to baseline, the mean score improvement maintained significance at 12 months follow-up in the STN (0.69 ± 0.79; 71%, *p* < 0.001) and the GPi (0.81 ± 0.63; 65%, *p* < 0.001) groups. In the between-group comparisons, while the STN group saw a greater improvement in AT at 6 months (*p* = 0.005), the 12 months comparisons were not significant (*p* = 0.301) (Figure 2).

The mean baseline RT scores for STN and GPi groups were 2.68 (±1.05) and 2.26 (±1.15), respectively. Compared to baseline, the mean score and percentage improvement at 6 months follow-up was significant for STN (0.86 ± 1.02; 68%, *p* < 0.001) and GPi (1.13 ± 1.08; 50%, *p* < 0.001) groups. At 12 months follow-up, the mean scores continued to further improve in the STN group (0.80 ± 0.13, 70%, *p* < 0.001) as well as in the GPi group (0.88 ± 0.19; 61%, *p* < 0.001). There were no between group differences at 6 months (*p* = 0.23) and 12 months (*p* = 0.74) follow-up.

Given the sample size differences in the AT and rest tremor analyses, the Levene’s test for equality of variance was conducted between the STN and GPi groups for the 6- and 12-months comparison. There were no statistically significant differences found in variance between the two groups at the 6- and 12-month time points for the AT and rest tremor comparisons.

**FIGURE 3:**
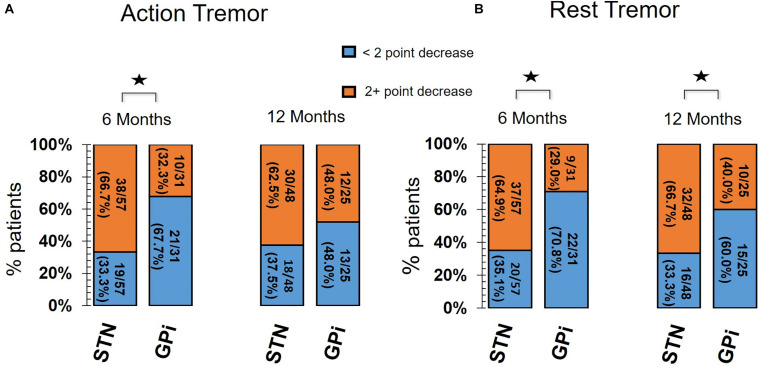
Bar chart for Optimal Responders versus Suboptimal Responders of **(A)** action tremor and **(B)** rest tremor. Orange segment of each bar represents number of Optimal Responders with ≥2 point drop in tremor score compared to baseline (before surgery). Blue segment of each bar represents number of Suboptimal Responders with <2 point drop in tremor score compared to baseline. The percentage or proportion of patients who were either optimal or suboptimal responders to STN DBS and GPi DBS for action tremor and rest tremor are shown at 6 and 12 months follow-up. UPDRS part III item 20 (for arm contralateral to DBS) was used for assessment of rest tremor. UPDRS part III item 21 (for arm contralateral to DBS) was used for assessment of action tremor. The STN DBS group had significantly greater number of optimal responders compared to suboptimal responders in the GPi DBS group for action tremor at 6 months and rest tremor at 6 and 12 months. *Denotes a statistically significant difference between STN and GPi groups.

The percentage number of “optimal responders” for AT was significantly greater in the STN group at 6 months follow-up (χ2 = 9.6, *p* = 0.01) however, there was no difference between STN and GPi groups at 12 months follow-up (χ2 = 1.1, *p* = 0.23). By contrast, the percentage number of “optimal responders” for RT remained significantly higher in the STN group compared to the GPi group at 6 months (χ2 = 10.4, *p* = 0.01) and at 12 months (χ2 = 4.8, *p* = 0.03). In the regression analysis for the GPi group, baseline AT severity (i.e., a higher score) significantly predicted the optimal responder rate at 6 months (OR 13.1; *p* = 0.02), but not at 12 months and conversely baseline RT severity predicted the improvement rate at 12 months (OR 7.3; *p* = 0.01) and not at 6 months. In the STN group, baseline RT severity significantly predicted at 6 (OR 9.8; *p* = 0.001) and 12 (OR 11.4; *p* = 0.001) months, whereas there were no predictors identified for the AT outcomes. There was no evidence for effects of the other predictor variables. There were no variables with a VIF greater than 1.7 or a tolerance lower than 0.6. Thus we concluded there was no multicollinearity among the variables selected for the model. A summary of sub-group distribution between “optimal responders” and “suboptimal responders” are illustrated in Figure 3 and the components of the regression model can be seen in Supplementary Table 1.

### DBS Response for Bradykinesia and Rigidity: Comparisons Between Targets

The mean change of bradykinesia and rigidity are also depicted in Figure 2. For bradykinesia, STN DBS provided a 48 and 36% improvement while GPi DBS provided 30 and 32% improvement from baseline to 6 and 12 months, respectively. For rigidity, STN DBS provided a 53 and 43% improvement while GPi DBS provided 23 and 28% improvement from baseline to 6 and 12 months, respectively. STN DBS provided a greater decrease in bradykinesia and rigidity compared to GPi DBS at 6 months post-implantation (*p* < 0.001 and *p* = 0.025, respectively). There were no differences between the two groups at 12 months post-implantation.

### Adverse Events

The surgery-related and device-related adverse events (AEs) for STN and GPi are summarized in Table 2. The most common AEs were DBS lead hardware issues and hemorrhage. The majority of hardware issues were short or open circuits discovered during the post-operative programming period. None of the hemorrhages required acute surgical intervention and all patients improved with conservative management.

**TABLE 2:** Summary of adverse events at one year follow-up.

**STN**		**GPi**	
**Adverse event**	**Number of events**	**Adverse event**	**Number of events**
DBS lead hardware problem	2	DBS lead hardware problem	4
Infection	1	Infection	1
Intracranial hemorrhage	5	Intracranial hemorrhage	2
Lead migration	1		
Seizure	3		
Twiddler’s syndrome	1		
Total events (%)	13		7

*STN, subthalamic nucleus; GPi, globus pallidus internus. The number of events do not necessarily reflect unique patients as some patients experienced multiple adverse events and thus were counted in multiple columns.*

### DBS Programming Parameters

The anatomical coordinates for the active contacts relative to the mid-commissural point (MCP) for the STN group were 11.3 ± 2.1 (mm) lateral to midline; 3.3 ± 1.5 (mm) anterior to MCP; 4.6 ± 2.3 (mm) ventral to intercommissural plane. The anatomical coordinates for the GPi group were 20.8 ± 1.6 (mm) lateral to midline; 0.8 ± 1.5 (mm) anterior to MCP; 4.8 ± 1.3 (mm) ventral to intercommissural plane.

The stimulation settings (mean ± SD and range) at one-year follow-up after implantation for the STN group were voltage (2.65 ± 0.6, 1.0–4.0), pulse width (97.9 ± 22.1, 60–150), and frequency (156.6 ± 25.7, 100–200). The stimulation settings (mean ± SD and range) at one-year follow-up after implantation for the GPi group were voltage (2.7 ± 0.8, 1.0–4.0), pulse width (88.2 ± 14.7, 60–120), and frequency (162.4 ± 25.3, 130–210).

In the *t*-test comparisons of suboptimal responders vs. optimal responders for AT and RT, we found the z coordinate of the active contact for RT optimal responders in the STN was statistically more dorsal with t (Mostofi et al., 2019) = −2.4 (*p* = 0.02) compared to suboptimal responders. However, these findings were not significant when corrected for multiple comparisons. The remaining comparisons pertaining to AT in STN group and AT and RT in the GPi group were not significant.

**FIGURE 4:**
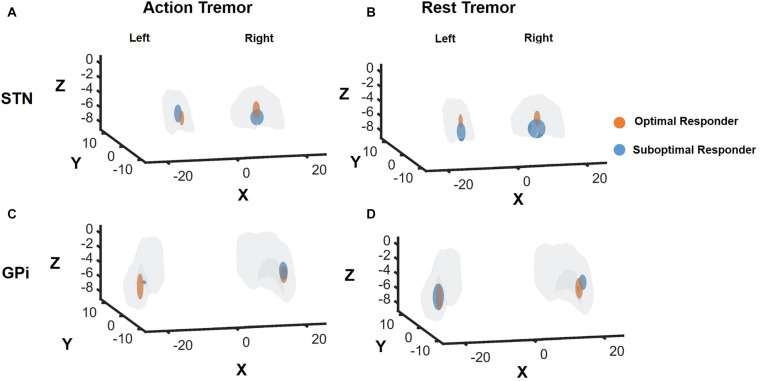
DBS contact locations in Optimal Responders versus Suboptimal Responders for STN and GPi DBS groups. Response for rest tremor and action tremor was individually analyzed. OR, responder, SR, suboptimal responder. “Optimal responders” were defined as patients experiencing >2 point decrease in the UDPRS Part III item 20 and 21 for rest and action tremor, respectively. “Suboptimal responders” were defined as <2 point decrease in the UDPRS Part III item 20 and 21 for rest and action tremor, respectively. The gray region in panels **(A,B)** represents the STN while the gray region in panels **(C,D)** represent the GPi. The orange bubble represents the “optimal responders,” and the blue bubble represents “suboptimal responders.” The size of the bubble is not electric field or volume of tissue activated. The size of the bubble represents the variance within each subgroup. While there were no significant differences between the two subgroups for action tremor control regardless of the target and rest tremor control with GPi DBS; however, rest tremor control with STN DBS suggested the optimal contact was slightly dorsal in location as seen along the *z*-axis.

The composite data can be seen in Figure 4 and individual coordinate data can be seen in Supplementary Table 2.

## Discussion

Action tremor in patients with PD can be particularly disabling as it can directly interfere with voluntary motor tasks. It has been hypothesized that the disruption of the cerebello-thalamo-cortical network may be involved in the underlying pathogenesis of tremor (Elble, 2013). Neuromodulation of the thalamus via VIM DBS evolved as a powerful treatment for essential tremor and PD tremor. VIM DBS does not, however, address other parkinsonian symptoms such as bradykinesia and rigidity. VIM DBS has been proposed as a possible target for AT in PD, however, our study findings indicate that STN and GPi DBS are both reasonable options to address many of the cases of moderate AT in patients with PD. In our cohort, one out of 88 patients required subsequent VIM DBS implantation suggesting that severe cases of PD tremor may benefit from VIM DBS to relieve the tremor adequately.

While previous studies have revealed that DBS therapy can effectively control tremor in PD, no study has examined the individual effects of DBS on AT and RT. Many surgical centers select STN as the target of choice for medication refractory PD tremor. Yet, a recent meta-analysis of randomized controlled trials did not detect a difference between STN and GPi in the tremor outcomes for patients with PD (Wong et al., 2018). It should be noted, however, the outcomes for the meta-analysis combined assessment of AT and RT, and there was no assessment of unique factors influencing tremor suppression. Here, we present a longitudinal single-center comparative analysis of a large dataset of PD DBS patients managed with both DBS targets. In the primary analysis there was no statistical difference and are consistent with several previous randomized controlled trials (Weaver, 2005; Weaver et al., 2012; Sako et al., 2014; Odekerken et al., 2016; Mansouri et al., 2017).

Although there were no overall differences in tremor outcomes between the two targets, we noted there was a temporal effect of DBS therapy on tremor. We observed that AT outcomes following STN and DBS were better than GPi at 6 months, but this effect disappeared by 12 months follow-up. This suggests that STN and DBS may be more effective in suppressing AT in the early postoperative period and that GPi DBS may require more time to appreciate the maximum benefit. There is increasing evidence to support the role of GPi in the pathogenesis of tremor. A functional imaging study revealed that the pathogenesis of PD tremor could be explained by a “dimmer – switch” model (Helmich et al., 2011). The efferent fibers from the GPi may trigger the tremor circuitry (analogous to a light switch) and dentato-rubro-thalamic (DRT) fibers may control the tremor intensity (analogous to a light dimmer). Hu et al. also found that GPi stimulation could paradoxically induce tremors in PD possibly due to stimulation spread to involve the pallido-thalamic outflow fibers (Hu et al., 2018). The temporal differences in DBS outcomes may be related to connectivity differences between the STN and GPi within the tremor circuit. Functional connectivity analyses suggest that the STN has both afferent and efferent connections with the cerebello-thalamo-cortical network whereas the GPi primarily has efferent connectivity (Helmich et al., 2012). When interpreted alongside physiology data, this has led to the proposal that the cerebello-thalamo-cortical network is the primary tremor generator and input from the basal ganglia contributes varying degrees of tremor modulation (Helmich et al., 2012, 2013; Helmich, 2013). Future DBS tractography studies, including larger samples of PD tremor will hopefully further elaborate on the role of GPi in RT and AT, specifically in the setting of PD. These studies will need to approach PD tremor from both the local nuclei effect as well as a network level perspective.

In the RT assessments, the between-group comparisons were not significant through 12 months of follow-up, however, one nuance in the data was that the percentage of “optimal responders” in the STN group was higher than GPi at both 6 and 12 months but there were no differences when AT outcomes was in consideration. These findings suggest that the RT circuitry is likely distinct and traverses the STN compared to the AT circuitry.

Analysis of postoperative lead localization and anatomical coordinates suggested that a dorsal STN location was more likely to optimally control RT, which may be related to modulation of DRT fibers traversing the posterior subthalamic region or fiber connectivity to the motor and premotor cortex (Plaha et al., 2006; Accolla et al., 2016; Eisinger et al., 2018; Mostofi et al., 2019). Analysis of AT outcomes, however, did not reveal a specific subregion within the STN. Also, we could not find a “sweet spot” for optimal tremor response in GPi, and this may have been due to small sample size.

Exploration of the factors that impacted the optimal vs. suboptimal tremor responders revealed a higher RT severity score at baseline increased the odds of prominent tremor suppression following DBS. While these results may suggest DBS has greater effects when the baseline tremor score is higher, we believe our findings are more possibly related to Weber’s law. According to the law the smallest discernible change in tremor amplitude is proportional to the initial baseline tremor amplitude (Elble, 2018). Thus, clinical raters are more likely to discern a 2-point drop (e.g., from a 4-to-2) in tremor score when collected using the tremor assessment item of the UPDRS as compared to a drop in someone with less baseline tremor (e.g., a 2-to-0).

Looking beyond tremor, previous target-based comparisons of DBS outcomes have revealed no major differences in the cardinal features of PD, including bradykinesia and rigidity when using either the STN or GPi target (Anderson et al., 2005). Like tremor outcomes, we observed a temporal difference in the DBS effects. We found that STN DBS led to superior rigidity and bradykinesia improvements in the early postoperative period compared to GPi DBS, but these differences were not sustained at one-year follow-up. This would again suggest that clinicians should be patient when programming the GPi target.

Our study had several limitations. First, the evaluation of AT was based on the UPDRS part III item 21 score, which combined postural and kinetic tremor. This score cannot differentiate between a re-emergent postural tremor and pure postural tremor. These differentiations can have important clinical implications as the underlying pathophysiology between the tremor subtypes are not identical (Jankovic et al., 1999; Dirkx et al., 2018). The score is also designed around a 4-point scale, limiting the resolution to more finely characterize important differences in tremor severity. Second, we recognize that medication intake can affect approaches to DBS programming and the total dosage requirement for dopaminergic medications may respond differently across various targets. However, levodopa responsiveness for tremor was given due consideration in our analysis and tremor scores were measured while off medications. Thus, the clinical measures in this study are unlikely to be affected by medication effect. Third, tremor assessments were not blinded, we did not include assessments longer than one year and we did not include VIM DBS for comparisons. Fourth, we did not assess the individual impact of RT and AT on the quality of life after DBS surgery. Fifth, as a common obstacle of longitudinal studies, a small percentage of our patients were lost to follow up at the 12-month time. All 88 patients in this study were retained at 6 months but 15 of the 88 patients in our study were lost to follow up by the 12-month visit. Finally, we do not have a gold standard test to determine co-pathology in patients within the cohort (PD plus essential tremor).

In conclusion, findings from this single-center cohort indicate that tremor control with STN and GPi DBS in PD is comparable regardless of whether RT and AT outcomes are individually assessed or combined. Clinicians should be aware that it may take longer to achieve a similar tremor outcome when utilizing the GPi target. The nuance of a possible higher rate of RT suppression with STN may suggest that the circuitry for RT traverses the STN and that the circuitry may be distinct from the AT network. Prospective larger multi-center studies with longer follow-up periods will be needed to confirm these findings.

## Data Availability Statement

Requests to access the datasets should be directed to aparna.shukla@neurology.ufl.edu.

## Ethics Statement

The studies involving human participants were reviewed and approved by University of Florida. The patients/participants provided their written informed consent to participate in this study.

## Author Contributions

JW and VV participated in study design, data acquisition, data analysis, and manuscript preparation. KN-F participated in data acquisition and data analysis. RE participated in data acquisition and data analysis and manuscript preparation. EL and AD participated in data acquisition. KF, AZ, and MO participated in manuscript preparation. AW participated in study design and manuscript preparation. All authors contributed to the article and approved the submitted version.

## Conflict of Interest

JW reports grant funding from the NIH (R25NS108939). KF reports grants from NIH, and other funding from Donnellan/Einstein/Merz Chair, during this study; grants and non-financial support from Medtronic, grants from St Jude, Functional Neuromodulation, and Boston Scientific, and grants and other funding from Neuropace. Additionally, KF has a patent US 8295935 B2 issued for a DBS cranial lead fixation device. MO serves as consultant for the National Parkinson’s Foundation, and has received research grants from the National Institutes of Health, National Parkinson’s Foundation, Michael J. Fox Foundation, Parkinson Alliance, Smallwood Foundation, Bachmann-Strauss Foundation, Tourette Syndrome Association, and UF Foundation. MO has previously received honoraria, but in the past >60 months has received no support from industry. MO has received royalties for publications with Demos, Manson, Amazon, Smashwords, Books4Patients, and Cambridge (movement disorders books). MO is an associate editor for New England Journal of Medicine Journal Watch Neurology. MO has participated in CME and educational activities on movement disorders (in the last 36 months) sponsored by PeerView, Prime, Quantia, Henry Stewart, and the Vanderbilt University. The institution and not MO receives grants from Medtronic, Abbvie, and ANS/St. Jude, and the PI has no financial interest in these grants. MO has participated as a site PI and/or co-I for several NIH, foundation, and industry sponsored trials over the years but has not received honoraria. AW reports grants from the NIH and has received grant support from Benign Essential Blepharospasm Research foundation, Dystonia coalition, Dystonia Medical Research foundation, National Organization for Rare Disorders and grant support from NIH (KL2 and K23 NS092957-01A1). AW reports receiving honoraria from Acadia, Cavion, Elsevier and MJFF in past; Participates as a co-I for several NIH, foundation, and industry sponsored trials over the years but has not received honoraria. The remaining authors declare that the research was conducted in the absence of any commercial or financial relationships that could be construed as a potential conflict of interest.

## Supplementary Material

Supplementary Table 1 Logistic Regression Analyses for Rest and Action Tremor Scores at 6 and 12 months (GPi and STN).

Supplementary Table 2 The mean of coordinates for optimal contact in the two subgroups of responders.
