# Supplementary material for: STN Versus GPi Deep Brain Stimulation for Action and Rest Tremor in Parkinson’s Disease
Source: Front Hum Neurosci. 2020 Oct 23;14:578615. doi: 10.3389/fnhum.2020.578615 (PMC7651783; doi:10.3389/fnhum.2020.578615)
Supplement: Supplementary Table 1 — Logistic Regression Analyses for Rest and Action Tremor Scores at 6 and 12 months (GPi and STN). [file Table_1.docx]

Supplementary table 1: Logistic Regression Analyses for Resting and Action Tremor Scores at 6 and 12 months (GPi DBS and STN DBS)

|  | Outcome | Predictor | *B* | *SE* | *Wald* | *df* | *p-value* | *Odds Ratio* | 95% CI |
| --- | --- | --- | --- | --- | --- | --- | --- | --- | --- |
| GPi |  |  |  |  |  |  |  |  |  |
|  | Rest Tremor,  6 months | Baseline Rest | 0.79 | 0.60 | 1.74 | 1 | 0.19 | 2.20 | [0.68, 7.10] |
|  |  | Rest Levodopa (di) | 3.00 | 1.86 | 2.62 | 1 | 0.11 | 20.12 | [0.529, 765.18] |
|  |  | Age | 0.05 | 0.06 | 0.53 | 1 | 0.47 | 1.05 | [.93, 1.18] |
|  |  | Disease Duration | 0.14 | 0.11 | 1.54 | 1 | 0.22 | 1.15 | [0.92,1.44] |
|  |  | Baseline Motor | -0.02 | 0.04 | 0.20 | 1 | 0.66 | 0.98 | [0.90,1.07] |
|  |  | Total Levodopa (di) | -1.02 | 1.29 | 0.63 | 1 | 0.43 | 0.36 | [0.03,4.52] |
|  |  | Constant | -7.78 | 4.76 | 2.67 | 1 | 0.10 | 0.00 |  |
|  |  |  |  |  |  |  |  |  |  |
|  | Rest Tremor,  12 months | Baseline Rest | 2.00 | 0.82 | 5.89 | 1 | **.015*** | 7.36 | [1.47, 36.89] |
|  |  | Rest Levodopa (di) | -0.56 | 1.33 | 0.18 | 1 | 0.68 | 0.57 | [0.04, 7.71] |
|  |  | Age | -0.05 | 0.10 | 0.23 | 1 | 0.63 | 0.96 | [0.79, 1.15] |
|  |  | Disease Duration | -0.08 | 0.12 | 0.52 | 1 | 0.47 | 0.92 | [0.73, 1.15] |
|  |  | Baseline Motor | 0.07 | 0.06 | 1.38 | 1 | 0.24 | 1.07 | [0.96, 1.19] |
|  |  | Total Levodopa (di) | -2.47 | 1.41 | 3.07 | 1 | 0.08 | 0.09 | [0.01, 1.34] |
|  |  | Constant | -2.89 | 5.13 | 0.32 | 1 | 0.57 | 0.06 |  |
|  |  |  |  |  |  |  |  |  |  |
|  | Action Tremor,  6 months | Baseline Action | 2.58 | 1.10 | 5.45 | 1 | **.020*** | 13.18 | [1.51, 114.73] |
|  |  | Action Levodopa (di) | -0.53 | 1.09 | 0.24 | 1 | 0.63 | 0.59 | [0.07, 4.99] |
|  |  | Age | -0.03 | 0.07 | 0.21 | 1 | 0.65 | 0.97 | [0.85, 1.11] |
|  |  | Disease Duration | 0.05 | 0.09 | 0.24 | 1 | 0.62 | 1.05 | [0.87, 1.26] |
|  |  | Baseline Motor | 0.04 | 0.04 | 1.09 | 1 | 0.30 | 1.04 | [0.96, 1.13] |
|  |  | Total Levodopa (di) | 0.29 | 1.13 | 0.06 | 1 | 0.80 | 1.33 | [1.51, 114.73] |
|  |  | Constant | -6.96 | 4.84 | 2.07 | 1 | 0.15 | 0.00 |  |
|  |  |  |  |  |  |  |  |  |  |
|  | Action Tremor,  12 months | Baseline Action | 5.58 | 3.36 | 2.76 | 1 | 0.10 | 265.35 | [0.37,192274.69] |
|  |  | Action Levodopa (di) | 6.33 | 3.52 | 3.22 | 1 | 0.07 | 558.31 | [0.56, 556877.69] |
|  |  | Age | -0.29 | 0.21 | 1.81 | 1 | 0.18 | 0.75 | [0.49, 1.14] |
|  |  | Disease Duration | -0.58 | 0.33 | 3.13 | 1 | 0.08 | 0.56 | [0.29, 1.06] |
|  |  | Baseline Motor | 0.26 | 0.21 | 1.55 | 1 | 0.21 | 1.30 | [0.86, 1.95] |
|  |  | Total Levodopa (di) | 0.26 | 1.66 | 0.03 | 1 | 0.87 | 1.30 | [0.05, 33.58] |
|  |  | Constant | -2.84 | 7.15 | 0.16 | 1 | 0.69 | 0.06 |  |
| STN |  |  |  |  |  |  |  |  |  |
|  | Rest Tremor,  6 months | Baseline Rest | 2.16 | 0.69 | 9.87 | 1 | **.002**** | 8.69 | [2.26, 33.47] |
|  |  | Rest Levodopa (di) | -0.81 | 1.22 | 0.44 | 1 | 0.51 | 0.45 | [0.04, 4.82] |
|  |  | Age | 0.05 | 0.04 | 1.24 | 1 | 0.27 | 1.05 | [0.97, 1.14] |
|  |  | Disease Duration | 0.08 | 0.10 | 0.70 | 1 | 0.40 | 1.08 | [0.89, 1.30] |
|  |  | Baseline Motor | 0.01 | 0.04 | 0.09 | 1 | 0.76 | 1.01 | [0.93, 1.10] |
|  |  | Total Levodopa (di) | 1.35 | 1.12 | 1.44 | 1 | 0.23 | 3.85 | [0.43, 34.85] |
|  |  | Constant | 2.16 | 0.69 | 9.87 | 1 | 0.00 | 8.69 |  |
|  |  |  |  |  |  |  |  |  |  |
|  | Rest Tremor,  12 months | Baseline Rest | 2.63 | 0.78 | 11.42 | 1 | **.001**** | 13.85 | [3.02, 63.57] |
|  |  | Rest Levodopa (di) | -1.17 | 1.38 | 0.71 | 1 | 0.40 | 0.31 | [0.02, 4.66] |
|  |  | Age | 0.00 | 0.05 | 0.01 | 1 | 0.95 | 1.00 | [0.92, 1.09] |
|  |  | Disease Duration | 0.05 | 0.10 | 0.29 | 1 | 0.59 | 1.06 | [0.87, 1.28] |
|  |  | Baseline Motor | 0.02 | 0.05 | 0.13 | 1 | 0.72 | 1.02 | [0.93, 1.12] |
|  |  | Total Levodopa (di) | 0.22 | 1.15 | 0.04 | 1 | 0.85 | 1.24 | [0.13, 11.83] |
|  |  | Constant | -6.98 | 4.46 | 2.45 | 1 | 0.12 | 0.00 | [3.02, 63.57] |
|  |  |  |  |  |  |  |  |  |  |
|  | Action Tremor,  6 months | Baseline Action | 1.34 | 0.71 | 3.56 | 1 | 0.06 | 3.80 | [0.95, 15.19] |
|  |  | Action Levodopa (di) | 1.14 | 0.77 | 2.18 | 1 | 0.14 | 3.12 | [0.69, 14.12] |
|  |  | Age | -0.02 | 0.03 | 0.50 | 1 | 0.48 | 0.98 | [0.91, 1.04] |
|  |  | Disease Duration | -0.08 | 0.07 | 1.26 | 1 | 0.26 | 0.93 | [0.81, 1.06] |
|  |  | Baseline Motor | -0.04 | 0.04 | 1.14 | 1 | 0.29 | 0.96 | [0.89, 1.03] |
|  |  | Total Levodopa (di) | 0.39 | 0.91 | 0.18 | 1 | 0.67 | 1.47 | [0.25, 8.69] |
|  |  | Constant | 0.65 | 3.01 | 0.05 | 1 | 0.83 | 1.91 |  |
|  |  |  |  |  |  |  |  |  |  |
|  | Action Tremor,  12 months | Baseline Action | 0.82 | 0.58 | 1.96 | 1 | 0.16 | 2.26 | [0.72, 7.07] |
|  |  | Action Levodopa (di) | -1.21 | 0.78 | 2.40 | 1 | 0.12 | 0.30 | [0.06, 1.38] |
|  |  | Age | 0.04 | 0.03 | 1.62 | 1 | 0.20 | 1.04 | [0.98, 1.11] |
|  |  | Disease Duration | -0.01 | 0.07 | 0.01 | 1 | 0.92 | 0.99 | [0.88, 1.13] |
|  |  | Baseline Motor | 0.00 | 0.03 | 0.00 | 1 | 0.97 | 1.00 | [0.94, 1.07] |
|  |  | Total Levodopa (di) | 0.36 | 0.81 | 0.20 | 1 | 0.66 | 1.44 | [0.29, 7.08] |
|  |  | Constant | -3.29 | 2.82 | 1.36 | 1 | 0.24 | 0.04 |  |

* Indicates significance at *p* <.05. ** Indicates significance at *p* <.01.
