# Supplementary material for: STN Versus GPi Deep Brain Stimulation for Action and Rest Tremor in Parkinson’s Disease
Source: Front Hum Neurosci. 2020 Oct 23;14:578615. doi: 10.3389/fnhum.2020.578615 (PMC7651783; doi:10.3389/fnhum.2020.578615)
Supplement: Supplementary Table 2 — The mean of coordinates for optimal contact in the two subgroups of responders. [file Table_2.docx]

|  |  | Suboptimal Responder | | | Optimal Responder | | |
| --- | --- | --- | --- | --- | --- | --- | --- |
|  |  | X | Y | Z | X | Y | Z |
| Action Tremor | STN | 11.4 ± 2.2 | - 3.0 ± 1.2 | - 4.7 ± 1.9 | 10.8 ± 1.5 | - 3.6 ± 1.5 | - 4.5 ± 2.1 |
|  | GPi | 20.2 ± 1.5 | 0.6 ± 2.0 | - 4.1 ± 1.7 | 20.8 ± 1.5 | 1.0 ± 1.0 | - 4.8 ± 2.1 |
| Rest Tremor | STN | 11.1 ± 2.3 | - 3.7 ± 1.7 | - 5.5 ± 1.9* | 11.0 ± 1.5 | - 3.2 ± 1.2 | - 4.1 ± 2.0* |
|  | GPi | 20.9 ± 1.7 | 0.8 ± 1.6 | - 4.2 ± 1.9 | 21.0 ± 1.7 | 0.8 ± 1.4 | - 4.9 ± 2.0 |

**Supplemental table 2:** The mean of coordinates for optimal contact in the two subgroups of responders

* indicates t (33) = - 2.4, p = 0.02. All other comparisons were not significantly different. Note that all X-values were converted to absolute values to collapse laterality.
